# Is Deep Acting Prevalent in Socially Responsible Companies? The Effects of CSR Perception on Emotional Labor Strategies

**DOI:** 10.3389/fpsyg.2019.00308

**Published:** 2019-02-18

**Authors:** Se Hyung Oh, Yein Hwang, Hwayoung Kim

**Affiliations:** School of Business, Hanyang University, Seoul, South Korea

**Keywords:** CSR perception, deep acting, surface acting, moral identification, affective organizational commitment

## Abstract

This study examines the relationship between corporate social responsibility (CSR) perception and emotional labor strategies, and the effects of the interaction between CSR perception and moral identification on emotional labor strategies via affective organizational commitment. Our data from 352 frontline employees in the service industry show the main effect of CSR perception on emotional labor strategies. We find that CSR perception is positively (negatively) related to deep acting (surface acting). Affective organizational commitment mediates the relationship between CSR perception and surface acting but not between CSR perception and deep acting. Moral identification moderates the effects of CSR perception on surface acting through affective organizational commitment. This paper reveals that the employees’ views on their organization’s social responsiveness and morality affect their emotional labor strategies.

## Introduction

Service workers often confront emotional challenges. Although employees frequently encounter demanding or even aggressive customers, they need to display the organizationally required emotions irrespective of their inner feelings. Presenting organizationally mandated emotions in this way is called emotional labor (Hochschild, 1983). To meet these emotional display rules and norms, employees usually engage in two types of emotional labor strategies: deep acting and surface acting. Deep acting is regulating the inner feelings to match the required emotional displays, whereas surface acting is superficially displaying the required feelings without modifying one’s inner feelings (Hochschild, 1983). Deep acting is considered to be a better emotional display strategy than surface acting since the resulting emotional expressions are perceived as authentic by customers (Grandey, 2003).

In the early studies of emotional labor, scholars have focused on the influence of organizations. In the seminal work of Hochschild (1983), emotional labor is seen as an occupational requirement which is a mean to achieve organizational objectives. In another early influential work by Rafaeli and Sutton (1987), emotional work is described as the behaviors obligated by organizations. Including these two studies, the focus in early works in emotional labor was the effects of organizational practices (e.g., selection, training, performance evaluation, socialization, culture, etc.) on service employees (e.g., Rafaeli and Sutton, 1987; Van Maanen and Kunda, 1989; Ashforth and Humphrey, 1993). However, since the early works, most emotional labor studies have focused on the effects of individual traits and dyadic relationships (see Pugh et al., 2013; Grandey and Gabriel, 2015). As a result, relatively little has been known about the effects of organizational-level factors on emotional labor (Pugh et al., 2013).

One important way to investigate the effects of organizational factors is to explore employees’ perceptions about the organization. Micro organizational behavior researchers have been using this way (e.g., Moorman et al., 1998; DeConinck, 2010; Alfes et al., 2013; Rupp et al., 2018). Although perceptions are different from objective organizational reality, they are more proximal predictors of individual attitudes and behaviors (e.g., Khilji and Wang, 2006; Nishii et al., 2008). Therefore, perceptions about the organization are a key to understand how organizational factors influence employee minds and behaviors.

This study investigates the effects of employees’ perceptions about their employer’s involvement in corporate social responsibility (CSR) activities, called CSR perception (Rupp et al., 2013), on their emotional labor strategies. Drawing on social identity theory (Tajfel and Turner, 1986), we expect that the positive perceptions and emotions about the employer generated from CSR perception induce strong motivation to engage in genuine rather than superficial emotional labor strategies. Through examining this hypothesis, this study intends to reveal that employees’ evaluation of the employer is an important determinant of emotional labor strategies.

Another objective of this study is to show the individual concerns about their employers’ morality can be a boundary condition of the effects of CSR perception. Employees’ CSR perception reflects the employer’s involvement in ethical and philanthropic activities (Maignan and Ferrell, 2000). Therefore, CSR perception would affect employees with high desire to belong to an ethical organization (May et al., 2015) more strongly than those with low desire. Through testing this proposition, we aim to show how employees’ moral desires interact with the employers’ perceived involvement in morally right activities (CSR) to affect the employees’ emotional labor strategies. The results of this study thus will demonstrate the importance of employer’s morality and employees’ moral desires on frontline service workers’ effectiveness and morale.

The current study fills the theoretical gap in both emotional labor and CSR literature. As mentioned, there is a lack of studies about the influence of organizational factors in the emotional labor literature (Pugh et al., 2013). Through investigating frontline service employees’ emotional and behavioral reactions to their perceptions about the employer, this study will provide insight toward the better understanding of the organization-level influences on employee emotional labor. This study will also contribute to CSR literature. Different from emotional labor research, CSR studies have mainly focused on the macro-level of analysis (Glavas, 2016) although CSR is a construct bridging macro and micro levels (Lindgreen and Swaen, 2010). Consequently, little has been known about how CSR directly influences employee behaviors (Glavas and Kelley, 2014). Through examining the effects of CSR perceptions on frontline employees, this study expands the current knowledge of mediation mechanism between corporate CSR activities and employee outcomes.

## Theoretical Background And Hypotheses

Corporate social responsibility refers to all company activities demonstrating the inclusion of social and environmental concerns in business operations, and in interactions with stakeholders, also according to the ambition levels of corporate sustainability (van Marrewijk, 2003). CSR has gained significant attention from researchers and practitioners (Aguinis and Glavas, 2012; Gond et al., 2017) because it generates various types of positive organizational outcomes such as corporate credibility in product (Lin et al., 2012), enhanced financial performances (Orlitzky et al., 2003), and positive relationships with customers (Matute-Vallejo et al., 2011). These positive effects are mainly due to enhanced organization’s image and attractiveness for external stakeholders (e.g., customers, shareholders, and society) (Minor and Morgan, 2011).

Researchers have also found that CSR activities induce positive outcomes from internal stakeholders. For example, Jones (2010) actually found that volunteering program through which employees perform community service enhances employee organizational citizenship behaviors (OCBs), and in-role performance. Turker (2009) also found that CSR were the significant predictors of employees’ organizational commitment. CSR activities for employees such as providing career growth opportunities or family-friendly policies affected the level of employees’ organizational commitment (Turker, 2009). Glavas and Piderit (2009) revealed that CSR induced positive employee outcomes such as engagement and creativity. These results confirm Rupp et al. (2006) proposition that CSR activities can trickle down to affect employees’ subsequent attitudes and behaviors in a positive way.

Despite the growing number of studies and interests on the effects of CSR activities on incumbent employees, due to the lack of the investigations about the mediational processes (Ng et al., 2018), how the macro-level CSR activities affect employees’ attitudes and performances are still vastly unknown (Glavas, 2016; Aguinis and Glavas, 2017). As a way of overcoming this theoretical problem, the concept of CSR perception is getting increasing attention from researchers. CSR perception is “employees’ perceptions of the extent to which their employer engages in various CSR activities” (Rupp et al., 2018, p. 560). It reflects how employees make sense of their organizations’ CSR activities and help explain the process of the individual-level consequences of CSR activities (Aguilera et al., 2007). Similar with objective CSR, CSR perception produces various types of positive employee outcomes such as identification (Kim et al., 2010), engagement (Caligiuri et al., 2013), performance (Jones, 2010), and creativity (Hur et al., 2016). Compared with objective CSR activities, CSR perception actually better predicted employee behaviors such as job satisfaction and turnover intentions (Valentine and Fleischman, 2008; Valentine and Godkin, 2016). It is because perception is a more immediate predictor of the employee attitudes and behaviors than the objective organizational reality (Rupp et al., 2013).

Our first hypothesis deals with the relationship between service employees’ CSR perceptions and their emotional labor strategies. Social identity theory maintains that an individual’s self-description is significantly influenced by the membership of social groups (Tajfel and Turner, 1986). According to this theory, because of self-enhancement needs, if a group is perceived as having characteristics positively distinguishing it from other groups, members increasingly identify with the group and accept its rules and norms (Tajfel and Turner, 1986; Ashforth and Mael, 1989). More importantly, when individuals strongly identify with a group, they are motivated to engage in behaviors that are beneficial for the group because they tend to identify the group’s wellbeing with their own (Tajfel and Turner, 1986).

Deep acting involves the motivation to alter the inner feelings to conform to the organization’s rules and norms (Gosserand and Diefendorff, 2005). This motivation is enhanced when employees like their organization (Mishra et al., 2012), accept its emotional display rules and norms (Gosserand and Diefendorff, 2005), and identify its success with their own (Ashforth and Humphrey, 1993). CSR activities enhance an organization’s image and attractiveness not only for external stakeholders (e.g., customers, shareholders, society) but also for incumbent employees (Rupp et al., 2006; Brammer et al., 2007; De Roeck et al., 2014). This means that if employees perceive that their employer actively engages in CSR activities, they increasingly identify with it and accept its rules and norms because of the employer’s enhanced images and attractiveness. Consequently, employees are motivated to match their inner feelings with the organization’s emotional display rules and norms to provide good customer service, enhancing the organization’s success. Based on this reasoning, we presume that employees’ CSR perceptions about their organizations are positively related to their deep acting.

Different from deep acting, surface acting reflects employees’ disengagement from the organizational norms and rules (Ozcelik, 2013). When employees are dissatisfied with their organization, they tend to dissociate themselves from it (Farrell, 1983). This type of employee is typically demotivated and follows organizational rules and norms superficially (Farrell, 1983). However, as mentioned, CSR perception increases employees’ satisfaction and identification with their organization. CSR perception also motivates employees to regulate their emotions to follow the organization’s emotional display rules and norms. Hence, we predict that employees’ CSR perceptions about organizations are *negatively* related to their surface acting.

H1: Employees’ CSR perceptions are positively related to their deep acting.

H2: Employees’ CSR perceptions are negatively related to their surface acting.

The next set of hypotheses deals with the mechanism of the influence of CSR perception on emotional labor strategies. In this study, we argue that affective organizational commitment is an important mediator between CSR perception and emotional labor strategies. Affective organizational commitment refers to the employee’s emotional attachment to, identification with, and involvement in the organization (Mowday et al., 1979). Affective commitment positively affects important employee outcomes such as job satisfaction (Boles et al., 2007), OCB (Ng and Feldman, 2011), and in-role performance (Meyer et al., 2002).

As mentioned, when employees are attracted to and proud of their organization, they are more likely to identify with and affectively committed to it (Ashforth and Mael, 1989; Brammer et al., 2007). This increased affective commitment, in turn, enhances employees’ motivation to follow the organization’s rules and norms (Mowday et al., 1979). When employees perceive that their organization is socially responsible, they increasingly identify with and affectively commit to it because of the organization’s distinctive positive characteristics (Aguilera et al., 2007; Brammer et al., 2007; Glavas and Kelley, 2014). This increased affective commitment would, in turn, intensify employees’ motivation to modify their inner feelings to provide good and professional customer service to support the achievements of the organization. In addition, the increased affective commitment due to CSR perception would reduce the inclination to provide superficial and shallow customer service. Hence, our hypotheses are as follows.

H3: The positive relationship between employees’ perceptions of their company’s CSR activities and their deep acting is mediated by their affective organizational commitment.

H4: The negative relationship between employees’ perceptions of their company’s CSR activities and their surface acting is mediated by their affective organizational commitment.

Recently, May et al. (2015, p. 682) developed a new construct called moral identification, which is defined as “the perception of oneness or belongingness associated with an organization that exhibits ethical traits (e.g., care, kindness, compassion), which also involves a deliberate concern of the membership with an ethical organization.” This construct reflects employees’ judgment about their organization’s morality as well as their desire to be part of an ethical organization. Employees with high moral identification tend to avoid unethical behaviors and have low turnover intentions when they believe that their organization is ethical (May et al., 2015).

In this study, we argue that moral identification moderates the relationship between CSR perception and affective organizational commitment. People with high moral identification care about the morality of their organizations and have a strong desire to be part of ethical organizations (May et al., 2015). Given that CSR activities are thought to be the ethical behaviors of companies (Carroll, 1991; Joyner and Payne, 2002), employees with high moral identification would show more positive attitudinal reactions to the CSR activities of their organizations. Empirical evidence shows that people with high moral concerns are more likely to react positively to organization activities focusing on helping external and internal stakeholders. For example, Rupp et al. (2013) found that a positive relationship between CSR perceptions and OCB was more pronounced among employees with high (vs. low) moral concerns (Rupp et al., 2013). Kolodinsky et al. (2010) also found that business students who have strong ethics of caring others showed more favorable attitude about CSR. Additionally, Wang et al. (2017) found that employees’ moral concerns amplified the positive effects of CSR perception on organizational identification. Consequently, we hypothesize that moral identification moderates the positive relationship between CSR perceptions and affective organizational commitment. More specifically, the influence of employees’ CSR perceptions on their affective organizational commitment would be stronger (weaker) when employees have high (low) moral identification.

H5: The positive effects CSR perception on affective organizational commitment is moderated by moral identification such that the effect is stronger (weaker) when moral identification is strong (weak).

**FIGURE 1:**
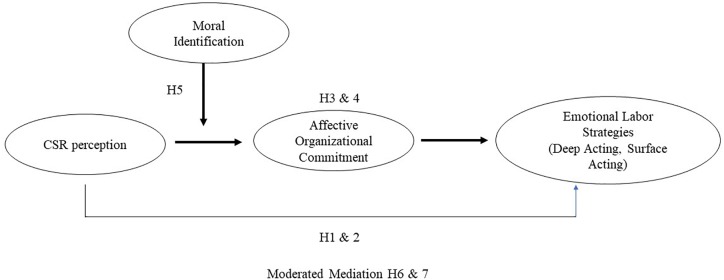
The moderated mediation model.

Thus far, we have hypothesized that (a) the relationship between CSR perception and emotional labor strategies is mediated by affective organizational commitment and that (b) the relationship between CSR perception and affective organizational commitment is moderated by moral identification. These hypotheses collectively suggest a moderated mediation model. As suggested, when employees perceive their organizations are socially responsible, they have enhanced affective organizational commitment, leading to increased deep acting and decreased surface acting. This indirect relationship will be stronger (vs. weaker) when the employees have strong (vs. weak) moral identification, given that CSR activities are thought to be the ethical behaviors of companies (Carroll, 1991; Joyner and Payne, 2002). Figure 1 depicts our research model. Our model predicts the first stage moderation mediation model where moral identification moderates the indirect effect of CSR perception on emotional labor strategies via affective organizational commitment.

H6: The positive indirect effect of CSR perception on deep acting via affective organizational commitment is moderated by moral identification such that the indirect effect is stronger (weaker) when moral identification is high (low).

H7: The negative indirect effect of CSR perception on surface acting via affective organizational commitment is moderated by moral identification such that the indirect effect is stronger (weaker) when moral identification is high (low).

## Methods

To test our hypotheses, we collected data from 352 service workers through an online research company that has more than one million panels. All respondents were frontline workers working as full-time regular employees in the organizations mostly in service (hotel, tourism, airline, etc.) (51%) and retail (department store, mall, etc.) (40%) industries. Rest of the respondents worked in hospitals, insurance companies, and transportation companies. Respondents’ average age was 37.8 years (*SD* = 9.8; min = 20; max = 72) and average tenure was 73.7 months (*SD* = 67.7; min = 1; max = 362). The average education level was between 2-year college and 4-year college. Over half (52%) of the respondents were women.

### Measures

We used a seven-point Likert scale ranging from 1 (strongly disagree) to 7 (strongly agree) throughout the questionnaire. The questionnaires were originally constructed in English but were translated into Korean. We used a translation and back-translation procedure (Brislin, 1980) to ensure the accuracy of the translation.

#### Corporate Social Responsibility Perception

Corporate social responsibility perception was measured with the five-item scale developed by Maignan and Ferrell (2000). A sample item is “Flexible company policies enable employees to better coordinate work and personal life.” The Cronbach’s alpha is 0.86.

#### Affective Organizational Commitment

Affective organizational commitment was measured with Meyer et al. (1993) six-item scale. However, exploratory factor analysis revealed that three items of the scale were double-loaded with CSR perception and these were excluded. The three remaining items used to measure affective organizational commitment are “I do not feel a strong sense of ‘belonging’ to my organization” (reverse coded), “I do not feel ‘emotionally attached’ to this organization” (reverse coded), and “I do not feel like ‘part of the family’ at my organization” (reverse coded). The Cronbach’s alpha is 0.94.

#### Deep Acting and Surface Acting

Deep acting and surface acting were measured by using the six-item scale developed by Brotheridge and Grandey (2002). Three items measured deep acting and the other three items measured surface acting. A sample item of deep acting is “when doing your job, how often do you try to actually experience the emotions you must show to customers?” The Cronbach’s alpha of deep acting is 0.86. A sample item of surface acting is “when doing your job, how often do you fake a good mood when interacting with customers?” The Cronbach’s alpha of surface acting is 0.81.

#### Moral Identification

Moral identification was measured by the five-item scale developed by May et al. (2015). This scale first requests respondents to imagine a person who is caring, compassionate, fair, friendly, generous, helpful, hardworking, honest, and kind. Then, it asks five questions based on the image of this person, such as “I strongly desire to be a member of an organization whose members have these characteristics.” The Cronbach’s alpha is 0.86.

### Data Analysis

In order to test Hypothesis 1 through 5, we conducted a series of regression analysis. The mediation hypotheses (Hypotheses 3 and 4) were examined by the methods suggested by Sobel (1982) and Baron and Kenny (1986). Finally, the moderated mediation hypotheses (Hypotheses 6 and 7) were tested through the bias-corrected bootstrapping techniques with 5,000 bootstrap samples using the PROCESS macro for SPSS (Hayes, 2013). This bootstrap technique involves the computation of the 95% bias-corrected bootstrap confidence intervals and it tests for a non-zero weight of the moderator in the indirect effect process. If the 95% bias-corrected confidence interval does not include zero, the moderation mediation is present.

## Results

### Preliminary Analyses

Table 1 presents the descriptive statistics, reliabilities, and correlations. CSR perception has a positive correlation with affective organizational commitment and moral identification. Affective organizational commitment and surface acting are negatively correlated.

**Table 1:** Means, standard deviations, reliabilities, and correlations among the study variables.

	*M*	*SD*	1	2	3	4	5	6	7	8	9	10
1. Age	37.81	9.76										
2. Sex	1.52	0.50	-0.154**									
3. Education	3.36	0.89	-0.143**	-0.232**								
4. Tenure	73.67	67.67	0.488**	-0.149**	-0.054							
5. Company size	4.88	1.64	-0.043	-0.050	-0.003	0.160**						
6. CSR perception	4.21	1.26	0.019	0.010	0.010	0.144**	0.285**	(0.860)				
7. Affective organizational commitment	3.98	1.42	0.032	0.046	-0.030	0.126*	-0.022	0.248**	(0.865)			
8. Surface acting	4.92	1.11	-0.127*	0.073	0.041	-0.064	0.098*	-0.078	-0.204**	(0.807)		
9. Deep acting	4.45	1.01	-0.017	-0.063	0.056	-0.044	0.113*	0.179**	-0.006	0.446**	(0.861)	
10. Moral identification	4.85	0.96	0.136**	-0.056	-0.014	0.157**	0.126*	0.412**	0.211**	0.016	0.229**	(0.862)


*N = 352. Numbers in parentheses on the diagonal are Cronbach’s alphas of the measures used in the study. ^∗^p ≤ 0.05, ^∗∗^p ≤ 0.01.*

We conducted a series of confirmatory factor analyses to verify the plausibility of our hypothesized factor structure. The proposed five-factor model demonstrated an acceptable fit to the data (χ^2^ = 458.89, df = 142, *p* < 0.001, CFI = 0.91, RMSEA = 0.08, TLI = 0.90, SRMR = 0.08). Furthermore, the five-factor model provided a significantly better fit than the other models, namely the four-factor model that combined CSR perception and moral identification into a single factor (χ^2^ = 1264.57, df = 146, *p* < 0.001, CFI = 0.69, RMSEA = 0.15, TLI = 0.73, SRMR = 0.09), the three-factor model that combined CSR perception, moral identification, and affective organizational commitment into a single factor (χ^2^ = 1725.66, df = 149, *p* < 0.001, CFI = 0.556, RMSEA = 0.18, TLI = 0.49, SRMR = 0.14), the two-factor model that merged CSR perception, moral identification, affective organizational commitment, and surface acting into a single factor (χ^2^ = 2260.89, df = 151, *p* < 0.001, CFI = 0.41, RMSEA = 0.208, TLI = 0.37, SRMR = 0.17), and the one-factor model that merged all five variables into a single factor (χ^2^ = 2581.2, df = 152, *p* < 0.001, CFI = 0.32, RMSEA = 0.22, TLI = 0.23, SRMR = 0.19). These results confirm the validity of our suggested factor structure.

When all data are self-reported and collected through the same questionnaire during the same period of time, common method variance is of specific concern. To assess the potential impact of common method variance, we conducted Harman’s single-factor test using the un-rotated principal component analysis and principal component analysis with varimax rotation was conducted. One-factor model of the un-rotated principal component analysis explained only 27.1% of total variance. The result of principal component analysis with varimax rotation showed that the first five eigenvalues were greater than 1.15. The five factors accounted for 73.5% of the total variance; however, no one single factor accounted for more than 25% of the variance, and the highest variance explained by any single factor was 17.6%, suggesting that the common method bias was mitigated.

### Hypothesis Testing

Table 2 reports the results of a series of regression analyses to test Hypotheses 1 to 5. In our analyses, we included age, sex, education, tenure, and company size. Sex affects individual differences in detecting emotional cues and managing own emotions (Domagalski, 1999). Age is influential on emotion control abilities (Kruml and Geddes, 2000) and tenure affects the experience of emotional dissonance (Hochschild, 1983; Kruml and Geddes, 2000). Education-level was found to affect emotional experience (Hamid and Cheng, 1996). Company size is influential on customers attitudes toward the company and its employees (Jarvenpaa et al., 1999), potentially affecting the frontline workers’ customer experience. All independent and control variables were mean-centered prior to entering the regression analyses.

**Table 2:** Regression test results.

	Dependent variables								
	Deep acting			Surface acting			Affective organizational commitment		
	Model 1	Model 2	Model 3	Model 4	Model 5	Model 6	Model 7	Model 8	Model 9
**Step 1. Control variables**									
Age	0.045	0.042	0.045	-0.132*	-0.131	-0.133*	-0.008	-0.030	-0.042
Sex	-0.115	-0.101	-0.112	0.174	0.187	0.191	0.120	0.135	0.111
Education	0.048	0.053	0.047	0.058	0.051	0.054	-0.031	-0.029	-0.043
Tenure	-0.058	-0.047	-0.056	0.026	0.031	0.035	-0.060	0.058	0.056
Company size	0.046	0.075*	0.044	0.086*	0.059	0.072	-0.090	-0.091	-0.094
**Step 2. Independent variables**									
CSR perception	0.136**		0.144**	-0.106*		-0.061	0.301**	0.241**	0.239**
Affective organizational commitment		0.004	-0.027		-0.161**	-0.148*			
Moral identification								0.196*	0.215*
Step 3. Interaction									
CSR perception × moral identification									0.135*
*R*^2^	0.049*	0.023	0.051*	0.044*	0.073**	0.077*	0.078**	0.092**	0.109*
Δ*R*^2^			0.028*			0.004			0.017*


*N = 352. ^∗^p < 0.05 ^∗∗^p < 0.01.*

**FIGURE 2:**
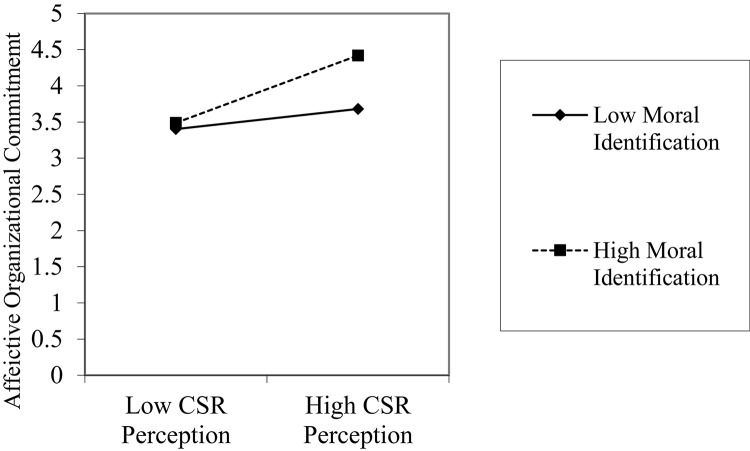
Moderating effects of moral identification.

Hypothesis 1 proposed that CSR perception is positively related to deep acting. As shown in model 1, after accounting for the effects of the control variables, we found that CSR perception significantly and positively affects deep acting (β = 0.136, *p* < 0.01), supporting Hypothesis 1. Hypothesis 2 predicted the negative influence of CSR perception on surface acting. Model 4 shows that CSR perception significantly and negatively affects surface acting (β = -0.106, *p* < 0.05), supporting Hypothesis 2. Hypotheses 3 and 4 predicted the mediating effects of affective organizational commitment between CSR perception and the two emotional labor strategies, deep acting (H3) and surface acting (H4). To test these hypotheses, we conducted a series of regression analyses suggested by Baron and Kenny (1986). As shown in model 1, the direct effect of CSR perception on deep acting is significant. Model 7 shows that CSR perception has a significant positive effect on the mediator variable, affective organizational commitment (β = 0.301, *p* < 0.01). However, as shown in model 2, the effect of the mediator variable, affective organizational commitment, on deep acting is not significant (β = 0.004, *p* = n.s.). In fact, compared with model 1, the effect of CSR perception on deep acting does not reduce even though affective organizational commitment is included in model 3. Therefore, Hypothesis 3 is not supported. Hypothesis 4 suggests the mediating effect of affective organizational commitment between CSR perception and surface acting. As seen in model 4, CSR perception has a significant negative effect on surface acting (β = -0.106, *p* < 0.05). Model 7 shows the CSR perception has a significant effect on affective organizational commitment (β = 0.301, *p* < 0.01). Model 5 shows that the mediator variable, affective organizational commitment, has a significant negative effect on surface acting (β = -0.161, *p* < 0.05). Finally, compared with model 4, the significant negative effect of CSR perception becomes insignificant when affective organizational commitment is added into model 6 (β = -0.061, *p* = n.s.), demonstrating the full mediating effect of affective organizational commitment. The result of the Sobel test (*Z* = -2.95, *p* < 0.01) confirms that CSR perception has an indirect effect on surface acting via affective organizational commitment. Therefore, Hypothesis 4 is supported. Hypothesis 5 is about the moderating effect of moral identification on the relationship between CSR perception and affective organizational commitment. Models 7, 8, 9 show the sequence of hierarchical regression analysis. In model 9, the interaction effect between CSR perception and moral identification on affective organizational commitment is significant (β = 0.135, *p* < 0.05). Figure 2 shows the patterns of interaction effects. Compared with employees with low moral identification, those with high moral identification showed a more positive relationship between CSR perception and affective organizational commitment. Therefore, Hypothesis 5 is supported.

Hypotheses 6 and 7 suggest that the indirect effects of CSR perception on the two emotional labor strategies (deep acting and surface acting) through affective organizational commitment are moderated by moral identification. We tested the moderated mediation model suggested in Hypotheses 6 and 7 by using the PROCESS macro (Hayes, 2013). Tables 3A,B summarize the results of the two moderated mediation tests. The 95% bias-corrected confidence intervals for the bootstrapped indirect effects at the two levels of the moderator, moral identification, were generated to test the significance of the conditional indirect effects. Table 3A shows the results of the test for Hypothesis 6. When moral identification is both high (+1 *SD*) and low (-1 *SD*), The 95% bias-corrected confidence intervals for the bootstrapped indirect effects from CSR perception to deep acting contain zero, indicating that moral identification does not moderate the indirect effect (see Table 3A). Therefore, Hypothesis 6 is rejected. Table 3B shows the results of the test for Hypothesis 7. When moral identification is low (-1 *SD*), the negative indirect effect of CSR perception is insignificant (*b* = -0.02, boot SE = 0.02, CI_95%_ = [-0.05, 0.02]). However, when moral identification is high (+1 *SD*), the negative indirect effect of CSR perception is significant (*b* = -0.05, boot SE = 0.03, CI_95%_ = [-0.11, -0.02]), showing that the significance of the indirect effects of CSR perception on surface acting via affective organizational commitment depends on the values of moral identification.

**Table 3A:** Conditional indirect effects of CSR perception on deep acting at values of moral identification.

Path	Moderator	Indirect effects	Boot SE	Boot LLCI	Boot ULCI
**Dependent variable: Deep acting**					
Simple path for low moral identification (-1 SD)	-0.96	0	0.01	-0.02	0.01
Simple path for high moral identification (+1 SD)	0.96	-0.01	0.02	-0.05	0.02


*95% bias-corrected confidence interval.*

**Table 3B:** Conditional indirect effects of CSR perception on deep acting at values of moral identification.

Path	Moderator	Indirect effects	Boot SE	Boot LLCI	Boot ULCI
**Dependent variable: Surface acting**					
Simple path for low moral identification (-1 SD)	-0.96	-0.02	0.02	-0.05	0.02
Simple path for high moral identification (+1 SD)	0.96	-0.05	0.03	-0.11	-0.02


*95% bias-corrected confidence interval.*

**FIGURE 3:**
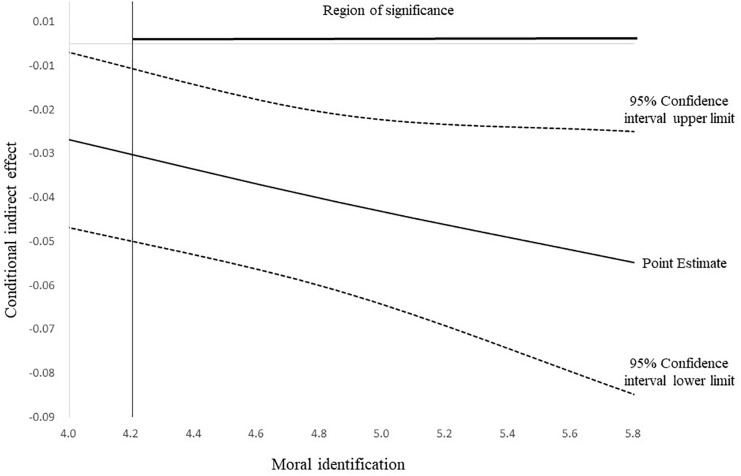
The conditional indirect effect of CSR perception on surface acting via affective organizational commitment with moral identification as a first-stage moderator. Dashed lines represent upper and lower confidence limits (95% bootstrap confidence intervals).

In order to probe the tested effect further, we used the Johnson-Neyman technique (Preacher et al., 2007; Hayes and Matthes, 2009). As seen in Figure 3, when moral identification is greater than 4.2, the negative indirect effect different from zero. This means that the negative effects of CSR perception on surface acting via affective organizational commitment are significant and amplified by moral identification when moral identification is greater than 4.2. Since the indirect effects of CSR perception on surface acting via affective organizational commitment are moderated by moral identification and the moderating effect occurs in the predicted direction, Hypothesis 7 is supported.

## Discussion

The purpose of this study was to examine whether employees’ perceptions about the employer’s involvement in CSR activities affect their emotional labor strategies. Another purpose was to explore the mechanism and a potential moderator of the suggested relationship. Our results show that CSR perception significantly affects employees’ emotional labor strategies. We found that CSR perception is positively associated with deep acting and negatively associated with surface acting. It seems that employees’ evaluation of their employer’s responsiveness to social matters and needs does affect their choice of emotional labor strategies. The perceived positive distinctiveness of the employer (CSR activities) seems to motivate employees to provide professional customer services with genuine emotional displays while reducing their inclination to serve customers superficially.

We also found that CSR perception significantly influences affective organizational commitment. Our data revealed that CSR perception has a significant positive impact on affective organizational commitment. It appears that employees have an increased sense of emotional attachment to the employer when they perceive that it is socially reputable. However, this increased affective organizational commitment accounts for the mechanism between CSR perception and surface acting only. Our results showed that affective organizational commitment fully mediates the relationship between CSR perception and surface acting. However, affective organizational commitment does not mediate the relationship between CSR perception and deep acting. Employees working for companies actively engaging in CSR activities enjoy the socio-emotional benefits from customers and society due to the company’s enhanced images and reputations (Minor and Morgan, 2011; Lii and Lee, 2012; Hur et al., 2014). This extrinsic rewards might account for the positive relationship between CSR perception and deep acting. Further investigation of the mediating mechanism of the CSR perception-deep acting relationship is worthwhile.

We found that moral identification moderates the positive effects of CSR perception on service employees’ organizational attitudes and emotional labor strategies. The positive effects of CSR perception on affective organizational commitment were stronger (weaker) when employees had a strong (weak) desire to be part of an ethical organization. Furthermore, moral identification moderated the indirect negative effect of CSR perception on surface acting via affective organizational commitment. Our data showed that the negative indirect effect of CSR perception on surface acting through affective organizational commitment is stronger (weaker) when employees have strong (weak) moral identification. It appears that employees who have a strong desire to be a member of an ethical organization tend to react to employer’s CSR activities more positively, resulting in a weaker tendency to provide superficial customer service.

### Theoretical Implications

This study offers several contributions to current emotional labor literature. First, it reveals a novel determinant of emotional labor strategies, CSR perception. Given that emotional labor is identity-challenging experiences (Ashforth and Humphrey, 1993), perceptions about an employer, which affects employees’ identity significantly (Hogg and Terry, 2000), should generate various types of emotional and behavioral consequences from service employees. This study provides one example. Our data show that how employees see the employers’ responsiveness to social needs (CSR perception) indeed had significant positive effects on deep acting and negative effect on surface acting. Similar with other studies about the effects of the perceptions about the employer (Duke et al., 2009; Janssen et al., 2010; Kraemer and Gouthier, 2014; Hur et al., 2016), enhanced organizational attitudes seem to account for the mediating mechanism. Future studies need to investigate more specific mediating mechanism between CSR perception and emotional labor strategies. For example, given that emotional labor has three components [emotion requirements (e.g., emotion display rules), emotion regulation (e.g., modifying feelings), and emotion performance (e.g., observable emotional expressions congruent with requirements)] (Grandey and Gabriel, 2015), how CSR perception affect each component of emotional labor is worthwhile to explore. Also how employee identity interacts with the three component of emotional labor is another important and interesting research topic.

This study provides implication on organizational morality-emotional labor relationship. CSR is an ethical organizational behavior in nature (Carroll, 1991). Therefore, the results of the current study imply that morality of the employer influences employee’s mindset and behaviors when they serve customers. Previous emotional labor researchers have found that the degree of organizational efforts on service ethics are positively associated with service employee emotional performances (e.g., appropriate emotional expression) (Ashforth and Humphrey, 1993). For example, Dietz et al. (2004) found that service workers provided enhanced customers services when excellent service is an important theme in an organization. Lam et al. (2010) also found that the service climate has a positive impact on positive emotional display and buffers negative influences from supervisors. Additionally, Pugh et al. (2013) pointed out that the organization-level utilization of human resource management (e.g., recruitment, training, performance monitoring, etc.) stressing customer service quality should have positive influences on employees’ emotional labor practices. This line of research is about the positive relationship between an organization’s efforts on service ethics and employees’ emotional performances. However, the current study suggests that not only the organization’s work- or service-related ethics but its ethics in general (e.g., responsibility about external and internal stakeholders in general) can generate important emotional labor consequences. Although there are emotional labor studies about the effects of ethics-related organizational factors such as organizational justice (Moliner et al., 2005) and perceived organizational support (Duke et al., 2009; Hur et al., 2013), we could not find any study explicitly focusing on the organizational morality and employee emotional labor. Given that individual and organizational morality is one of the major factors of employee identity, decision making and behaviors (Trevino et al., 2006), organizational morality is more likely to affect employee emotional performance. Our data provide initial evidence. Future researchers need to investigate more about the organizational morality-emotional labor relationship.

Finally, the current study provides implications on the conservation of resources (COR; Hobfoll, 1989) theory-based emotional labor studies. COR theory, originally introduced as a motivation theory, claims that “individuals strive to obtain, retain, protect, and foster those things that they value” (Hobfoll, 2011, p. 341). COR theory is one of the main theoretical frameworks in explaining the processes and consequences of emotional labor (Grandey and Gabriel, 2015). It suggests that involuntary emotional regulation to match the organization’s emotional requirements consumes psychological resources, resulting in emotional exhaustion (Brotheridge and Lee, 2002). It also suggests that gains in motivational resources such as task significance and social support can help service workers to prevent resource losses and enable them to cope with stresses from service works (Grandey and Diamond, 2010). There are many emotional labor studies based on COR theory (e.g., Chau et al., 2009; Grandey and Diamond, 2010; Goodwin et al., 2011; Bhave and Glomb, 2016) and most evidence fit well with the theory. The current study provides novel insight on this resource-based perspective. The results of this study imply that CSR perception can be a source of motivational resources that service workers consume when they interact with customers. Although we could not find any studies framing perceptions about employer as a source of resources, we noticed that there are several studies resonating with this idea. For example, Duke et al. (2009) demonstrated that perceived organizational support can provide a psychological buffer from the frustration and dissatisfaction experienced from service works in retail stores. Gouthier and Rhein (2011) showed that frontline employees’ pride about organization affects their motivation to commit to customer service positively. Kraemer and Gouthier (2014) also revealed that service workers’ organizational pride alleviate the emotional exhaustion and turnover intention. This line of research suggests that positive perceptions about the employer such as CSR perception provide psychological and motivational resources to cope with negative experiences from service works. Whether CSR perception actually works as other resources (e.g., helping employees to tolerate stresses and emotional exhaustion) is a worthwhile topic to investigate for future researchers.

### Managerial Implications

This study provides clear practical implications. First, it is important for service organizations to become socially responsible. Our results show that when employees see that their organizations have high responsibility on social matters and needs, their organizational attitudes become positive and their emotional performance toward customers is enhanced. CSR activities have long been considered to be only as a tool for public relations (Middlemiss, 2003; Kim and Park, 2011). However, practitioners should also consider them to be an instrument to enhance employee morale and organizational performance.

Second, management needs to understand the individual differences in the reaction to CSR activities. Indeed, CSR activities can generate both positive and negative impacts on the employees’ morale (McShane and Cunningham, 2012). Based on our results, we can speculate that individual differences such as moral identification could be one of the reasons. Therefore, for example, recruiting employees for CSR activities based on their individual differences in values and personalities should increase the positive consequences of CSR activities.

Finally, managers need to be concerned about employees’ emotional attachment to the organization. Our results suggest that employees’ affective organizational commitment, which rises with their CSR perceptions, plays an important role in service workers’ attitudes and emotional performance. Given that affective organizational commitment is the direct precursor of emotional labor strategies, a manager who wants to enhance employees’ emotional performance should care about their emotional attachment to the organization.

### Limitations and Future Research

The current study is not without limitations. First, our results are based on cross-sectional data. Although cross-sectional data are still widely used in emotional labor studies (e.g., Molino et al., 2016; Indregard et al., 2018a,b), using time-lagged data would increase the persuasiveness of the suggested causal relationships in this study. Future research would thus benefit from using time-lagged data to analyze the dynamics of employees’ CSR perceptions on their organizational attitudes and behaviors. Second, our data depend on self-reports. We used self-report data because all our variables deal with employees’ feelings, which are difficult for others to measure correctly. Although Harman’s single factor results showed that our data do not have a common method variance problem (Podsakoff et al., 2012), future studies can confirm our results by conducting a study collecting data from multi sources (e.g., customers, coworkers, etc.). Third, our data were collected only from Korean subjects. Since cultural orientation affects employees’ attitudes and behaviors in organizational settings (Hofstede and Hofstede, 2005), our collectivistic Koreans data may show different patterns from individualistic Westerner data. Further research using data from subjects with different cultural orientation is thus required to confirm the external validity of our results.

## Conclusion

This study examines the influence of employees’ CSR perceptions on their emotional labor strategies. Our results demonstrate that CSR perception encourages deep acting and discourages surface acting. Affective organizational commitment explains why CSR perception discourages the inclination to provide superficial customer services. Finally, employees’ desire to be a member of an ethical organization amplifies or diminishes the effect of CSR perception on surface acting via affective organizational commitment. This study suggests that management should be concerned about employees’ perceptions of an emotional attachment to the organization in order to enhance their emotional performance to customers.

## Ethics Statement

This study was carried out in accordance with the recommendations of Hanyang University Institutional Review Board with written informed consent from all subjects. All subjects gave written informed consent in accordance with the Declaration of Helsinki. The protocol was approved by the Hanyang University Institutional Review Board.

## Author Contributions

SO conceived the original idea, developed the theoretical models, and took the lead in writing the manuscript. YH helped developing theories and wrote theories for Hypotheses 1, 2, 3. HK carried out data analyses, interpreted the results, and wrote the “Materials and Methods” and “Results” section. SO, YH, and HK, all three of them, significantly contributed to manuscript.

## Conflict of Interest Statement

The authors declare that the research was conducted in the absence of any commercial or financial relationships that could be construed as a potential conflict of interest.
